# Sexual dysfunction during treatment with serotonergic and noradrenergic antidepressants: Clinical description and the role of the *5-HTTLPR*

**DOI:** 10.3109/15622975.2011.559270

**Published:** 2011-03-09

**Authors:** Jana Strohmaier, Stefan Wüst, Rudolf Uher, Neven Henigsberg, Ole Mors, Joanna Hauser, Daniel Souery, Astrid Zobel, Mojca Z Dernovsek, Fabian Streit, Christine Schmäl, Dejan Kozel, Anna Placentino, Anne Farmer, Peter Mcguffin, Katherine J Aitchison, Marcella Rietschel

**Affiliations:** 1 Central Institute of Mental Health, Division of Genetic Epidemiology in Psychiatry, Mannheim, Germany; 2 Medical Research Council (MRC) Social, Genetic, and Developmental Psychiatry Research Centre, Institute of Psychiatry, King's College London, UK; 3 Croatian Institute for Brain Research, Medical School, University of Zagreb, Croatia; 4 Aarhus University Hospital, Risskov, Denmark; 5 Laboratory of Psychiatric Genetics, Department of Psychiatry, Poznan University of Medical Sciences, Poland; 6 Laboratoire de Psychologie Médicate, Université Libre de Bruxelles and Psy Pluriel – Centre Européen de Psychologie Médicale, Belgium; 7 Department of Psychiatry, University of Bonn, Germany; 8 Educational and Research Institute Ozara, Ljubljana, Slovenia; 9 Institute of Psychobiology, University of Trier, Germany; 10 Institute of Public Health, Ljubljana, Slovenia; 11 Psychiatric Unit 23, Department of Mental Health, Spedali Civili Hospital and Biological Psychiatry Unit, Centro San Giovanni di Dio IRCCS-FBF, Brescia, Italy; 12 Division of Psychological Medicine and Psychiatry, Institute of Psychiatry, King's College London, UK

**Keywords:** Sexual dysfunction, *5-HTTLPR*, selective serotonin uptake inhibitors, escitalopram, nortriptyline

## Abstract

**Objectives:**

Sexual dysfunction (SD) is a frequently reported side-effect of antidepressant treatment, particularly of selective serotonin reuptake inhibitors (SSRIs). In the multicentre clinical and pharmacogenetic GENDEP study (Genome-based Therapeutic Drugs for Depression), the effect of the serotonin transporter gene promoter polymorphism *5-HTTLPR* on sexual function was investigated during treatment with escitalopram (SSRI) and nortriptyline (tricyclic antidepressant).

**Methods:**

A total of 494 subjects with an episode of DSM-IV major depression were randomly assigned to treatment with escitalopram or nortriptyline. Over 12 weeks, depressive symptoms and SD were measured weekly with the Montgomery-Asberg Depression Rating Scale, the Antidepressant Side-Effect Checklist, the UKU Side Effect Rating Scale, and the Sexual Functioning Questionnaire.

**Results:**

The incidence of reported SD after 12 weeks of treatment was relatively low, and did not differ significantly between antidepressants (14.9% escitalopram, 19.7% nortriptyline). There was no significant interaction between the *5-HTTLPR* and antidepressant on SD. Improvement in depressive symptoms and younger age were both associated with lower SD. The effect of age on SD may have been moderated by the *5-HTTLPR*.

**Conclusions:**

In GENDEP, rates of reported SD during treatment were lower than those described in previous reports. There was no apparent effect of the *5-HTTLPR* on the observed decline in SD.

## Introduction

Patients who receive antidepressants frequently report sexual dysfunction (SD), which may affect all phases of the sexual response cycle, i.e. desire, arousal, and orgasm. Estimates of medication-induced SD vary widely. Rates of between 10 and 90% have been reported, with higher rates for antidepressants which act on the serotonergic system (Werneke et al. 2006; Serretti and Chiesa 2009; Williams et al. 2010). SD rates of around 40% have been reported for the selective serotonin reuptake inhibitor (SSRI) escitalopram (Clayton et al. 2006b, 2007), although lower rates were reported in a review published in 2004 (Hirschfeld and Vornik 2004). Only one previous report has compared rates of SD during treatment with tricyclics and SSRIs. This retrospective study included a small male sample and compared SD rates during treatment with five tricyclic antidepressants (including nortriptyline) and three SSRIs (not including escitalopram). A higher rate of treatment-associated SD was observed during treatment with SSRIs (Hsu and Shen 1995). Research suggests that SSRIs cause dysfunction in all phases of the sexual response cycle, predominantly in the phases of desire and orgasm in males and arousal in females (Clayton et al. 2006a; Corona et al. 2009; Serretti and Chiesa 2009). TCAs have been found to have particular effects on desire and orgasm, although they may also affect other phases of the sexual cycle since they modify several neurotransmitters (Werneke et al. 2006). SD is known to decrease compliance with antidepressant treatment, and effective strategies for its management are therefore essential (Rothschild 2000; Montejo et al. 2001; Werneke et al. 2006).

Pharmacogenetic research has only recently begun to identify genetic markers that may predict response to antidepressant treatment (Lin and Chen 2008; Drago et al. 2009; Ising et al. 2009; Schosser and Kasper 2009; Uher et al. 2009b, 2010; Kato and Serretti 2010). These markers may facilitate the prediction of adverse events such as SD during antidepressant treatment (Bishop et al. 2009). It has been hypothesised that the development of SD during treatment with serotonergic antidepressants is moderated by variations in the serotonin transporter gene *(SLC6A4),* which encodes the serotonin transporter protein (5-HTT; Bishop et al. 2009). The polymorphism in *SLC6A 4* which has been most studied for association with SD to date is the *5-HTTLPR,* an insertion/deletion of 44 base pairs (bp) within the promoter region. An association has been reported between *5-HTTLPR* genotype and response to antidepressant treatment, in which carriers of the long allele show a better response than short allele homozygotes (Serretti et al. 2007; Huezo-Diaz et al. 2009). Long allele homozygotes have also been shown to experience more SD during SSRI treatment for depression (Bishop et al. 2009). Furthermore, an association has been found between the short allele and premature ejaculation (Ozbek et al. 2009), whereas Janssen et al. (2009) reported in s-carriers with premature ejaculation longer ejaculation times compared to long allele homozygotes. In a study of the treatment of premature ejaculation, long allele homozygotes showed a better SSRI treatment response than short allele carriers (Safarinejad 2010a).

Pharmacologically, SSRIs reduce the binding of serotonin to the serotonin transporter – a protein responsible for the transport of serotonin from the synaptic cleft into presynaptic serotonergic neurons – thereby increasing the concentration of synaptic serotonin (5-HT) and facilitating the modulation of post-synaptic receptors (Stahl 1998). This SSRI effect may additionally decrease the concentrations of dopamine and noradrenaline in the mesolimbic system by activating serotonin 5-HT_2_ receptors (Meltzer et al. 1979; Baldessarini and Marsh 1990; Done and Sharp 1992). Dopamine release within the mesolimbic system has been implicated as a major mechanism in sexual function (Bitran et al. 1988; Segraves 1989; Hull et al. 1999). Noradrenaline regulates sexual arousal (Lee and Pfaff 2008) and bupropion, a dopamine and noradrenaline reuptake inhibitor, has been reported to decrease SSRI-induced SD (Zisook et al. 2006; Safarinejad 2010b,c). However, the mechanisms through which SSRIs cause SD remain poorly understood (Segraves 2007; Perlis et al. 2009). SSRIs are currently the most commonly prescribed antidepressants. They have been reported to be highly effective in the treatment of depression and to cause fewer severe side effects than TCAs (Degner et al. 2004; Grohmann et al. 1999). It is therefore important to elucidate the association between SSRIs and SD.

The aim of the present study was to assess SD rates during treatment with escitalopram and nortriptyline, and to determine the possible influence of *5-HTTLPR* genotype on sexual function. The study was conducted within the context of a large European Commission funded multicentre study (Genome-based Therapeutic Drugs for Depression – GENDEP). Given previous findings of an association between *5-HTTLPR* genotype, SD, and treatment response during antidepressant treatment, we hypothesised that carriers of the *5-HTTLPR* long allele would experience a higher degree of SD during SSRI treatment than either short allele homozygotes or patients receiving TCAs.

## Methods

### Study design and sample

The GENDEP project is a multicentre pharmacogenetic study designed to compare the clinical and genetic determinants of therapeutic response to two antidepressants with contrasting primary modes of action – nortriptyline and escitalopram (http://gendep.iop.kcl.ac.uk/results.php). The study was partly randomised, i.e. patients were only randomised to treatment in the absence of any contraindication to either of the two study drugs. All subjects met ICD-10/DSM-IV criteria for a major depressive episode. Patients were treated with nortriptyline or escitalopram for a period of 12 weeks. Escitalopram was initiated at a dose of 10 mg daily, and this was increased to a target dose of 15 mg daily within the first 2 weeks. The dose could be further increased to 20 mg daily, or up to 30 mg when there was clinical agreement that a higher dose was indicated. Nortriptyline was initiated at 50 mg daily and titrated to a target dose of 100 mg daily within the first 2 weeks. The dose could be increased to 150 mg daily, or up to 200 mg when there was clinical agreement that a higher dose was indicated. Adjunct psychotropic medication was not allowed, with the exception of occasional hypnotics. Compliance was monitored weekly by self-reported pill count, and antidepressant plasma levels were measured at week 8. Written informed consent was obtained from all participants prior to inclusion. A detailed description of the GENDEP sample and treatment outcomes is provided elsewhere (Uher et al. 2009c). Only subjects who had been randomly allocated to treatment were included in the present study to exclude possible confounding between pre-treatment SD and treatment allocation. The final sample included 176 males (mean age 41.7 ± 12.5 years) and 318 females (mean age 42.4 ± 11.0 years). Forty-six males and 70 females discontinued the study medication before week 12 and were thus considered study drop-outs.

### Measures

Depression severity was measured weekly using three established scales: (i) the clinician-rated 10-item Montgomery-Asberg Depression Rating Scale (MADRS); (ii) the clinician rated 17-item Hamilton Rating Scale for Depression (HRSD-17); and (iii) the self-report 21-item Beck Depression Inventory (BDI). The MADRS provides the most accurate and internally valid reflection of depression severity (Uher et al. 2008). Joint factor analysis of the items of all three depression scales revealed three depressive symptom dimensions that provide a more detailed description of depression severity: (i) observed mood and anxiety, (ii) cognitive symptoms, and (iii) neurovegetative symptoms (Uher et al. 2008).

Sexual function was measured weekly with the Antidepressant Side-Effect Checklist (ASEC; Uher et al. 2009a). The ASEC is a self-report instrument that was designed to measure 21 adverse reactions to antidepressants. Item 12 rates problems with sexual function according to a four-point scale (absent, mild, moderate, severe). Each study participant was asked to complete the ASEC before commencing the study medication, and then weekly for 12 weeks. The ASEC item 12 was selected as the main outcome measure due to its weekly application and low rate of missing data. The UKU Side Effects Rating Scale (UKU) is a comprehensive semi-structured interview which assesses psychic and physical adverse reactions to psychotropic drugs (Lingjaerde et al. 1987). Five of the 48 UKU items (desire, erection, ejaculation, vaginal lubrication, orgasm) enquire specifically about SD according to a four-point scale. The Sexual Functioning Questionnaire (SFQ; Smith et al. 2002) is a 38-item self-rating questionnaire that measures current sexual functioning across various domains (libido, arousal, erection, ejaculation, vaginal lubrication, orgasm) according to a two-point scale. Higher scores indicate greater sexual dysfunction in the respective domain. In GENDEP, the *UKU* and the *SFQ* were administered at baseline and at study weeks 8 and 12 (Uher et al. 2009a).

### Genotyping and quality control

Blood samples were collected in ethylenediamine tetra-acetic acid and frozen. DNA was extracted using a standard procedure (Freeman et al. 2003). Short and long alleles of the *5-HTTLPR* were determined using polymerase chain reaction (PCR) with a previously described method (de Bakker et al. 2005).

### Statistical analyses

A repeated measures ANOVA was used to compare differences in the main outcome measure *(ASEC* item 12) at baseline and all subsequent study weeks between drop-outs and subjects who completed the entire protocol. In this analysis, study week was a within-subject factor and drop-out vs. non-drop-out and sex were between-subject factors. All subsequent ANOVAs were computed with study week as a within-subject factor and sex as a between-subject factor. For the influence of study medication, a repeated measures ANOVA was computed with study medication as a between-subject factor. In a subsequent step, change in depressive symptoms during the course of treatment (difference in MADRS summary scores and depressive symptom dimensions between baseline and week 12), age, and genotype status at the *5-HTTLPR* locus (s/s vs. s/1 vs. 1/1) were added to the model as further predictors.

To limit the number of statistical analyses, only the genetic association between the 5-HTTLPR and the main outcome measure (ASEC item 12) was tested. A further reason for focusing the genetic association analysis on ASEC item 12 was the high rate of missing data for the UKU and SFQ, which resulted in very low statistical power.

For the five individual UKU items and the SFQ subscales, similar repeated measures ANOVAs were performed as those carried out for the main outcome measure (ASEC item 12). Study week was entered as a within-subject factor and sex as a between-subject factor. The stepwise inclusion of study medication, age, and the change in depressive symptoms during the course of treatment (difference between MADRS summary score at baseline and week 12) were considered as further predictors. Due to a high rate of missing data for the SFQ, this analysis was restricted to data from baseline and study week 12.

All analyses were performed in SPSS version 17.0. Greenhouse-Geisser corrections were applied where appropriate, and only adjusted results are reported.

## Results

### Genotyping

Genotype data were available for 473 subjects. Genotype frequencies for the *5-HTTLPR* variant were 192 1/1 (0.41), 211 1/s (0.45), and 70 s/s (0.14). These are consistent with previously reported frequencies, and did not deviate significantly from Hardy-Wein-berg equilibrium (*P* = 0.33).

### Results obtained front the various sexual function rating scales

*Antidepressant Side-Effect Checklist*. Subjects who dropped out of the study did not differ significantly from those who completed the protocol in terms of the main outcome measure (ASEC item 12; main effect Group *F*_1;156_ = 0.064, *P* = 0.801; interaction Sex × Group *F*_1;156_ = 0.112, *P* = 0.738). The data of study drop-outs were therefore included in the analyses. The drop-out rate was linear, with around 10% of running subjects dropping out each week.

**Figure 1:**
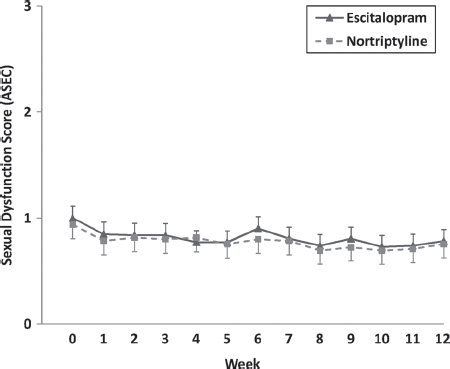
Time course of reported sexual dysfunction (*ASEC* item 12) over the 12-week study period for escitalopram and nortriptyline.

In both treatment groups, the majority of patients reported no, or only mild, SD according to ASEC item 12 (see Table I). Furthermore, a trend towards a decrease over time was observed (main effect Time *F*_4.477;158_ = 2.037, *P* = 0.08). Separate analyses for males and females revealed a significant main effect for Time in females only (*F*_5.437;98_ = 2.963, *P* = 0.01), with not even a trend being identified in males (*F*_2.466;60_ = 0.908, *P* = 0.423). The highest SD rates were reported at baseline, i.e. when the subjects were still medication free. On a descriptive level, this trend was more pronounced in females than in males, with 20.3% of females reporting severe SD at baseline and only 5.8% of males (see Discussion). There was no evidence that study medication influenced the overall severity or the time course of SD (main effect Medication *F*_1;156_ = 0.027, *P* = 0.869, interaction Time × Medication *F*_4.491;156_ = 0.226, *P* = 0.939; Figure 1).

**Table I:** Percentage of reported SD according to *ASEC* item 12.

	SD	Baseline	SW 1	SW 2	SW 3	SW 4	SW 5	SW 6	SW 7	SW 8	SW 9	SW 10	SW 11	SW 12
Males	None	53.8	61.5	61.2	62	64	59.1	59.8	60.3	58.9	58.8	62.1	65.6	65
	Mild	19.9	17.3	18.4	18.7	20.9	21.9	20.5	22.4	21.4	21.6	21.1	17.8	19
	Moderate	20.5	15.4	15.1	14.7	10.8	13.1	13.4	10.3	13.4	13.7	9.5	11.1	12
	Severe	5.8	5.8	5.3	4.7	4.3	5.8	6.3	6.9	6.3	5.9	7.4	5.6	4
Females	None	52.6	66.5	68.4	69.8	70.5	70.7	62.4	69.2	68.5	66.7	67.8	64.2	66.1
	Mild	11.7	10.2	9.6	10.1	8.1	7.9	11.4	7.1	9.3	10.4	8.7	11.4	9.8
	Moderate	15.5	10.2	7.4	7.1	7.8	7.5	13.1	9.8	12	11.5	11.5	13.6	12
	Severe	20.3	13	14.7	13.1	13.6	13.8	13.1	13.8	10.2	11.5	12	10.8	12

SW, study week.

**Figure 2:**
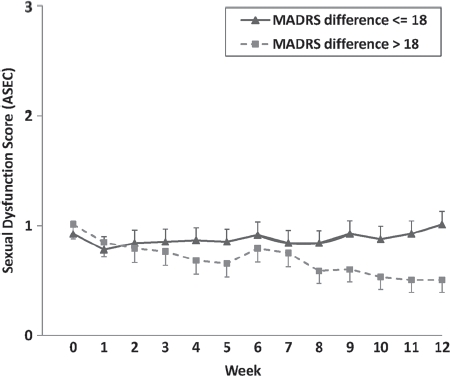
Time course of reported sexual dysfunction (*ASEC* item 12) over the 12-week study period according to the change in MADRS (Montgomery-Asberg Depression Rating Scale) in subjects with scores £18 points and >18 points.

An analysis was then performed to investigate whether the change in depressive symptoms (i.e. the difference between MADRS summary scores at baseline and at week 12) had any impact on SD. The ANOVA revealed a significant interaction effect of Time × MADRS (F_4__780_._154_ = 5.016,P< 0.001). This effect was controlled for medication and sex, although it was virtually identical without controlling for these variables. To provide a graphical illustration (Figure 2), a median split was performed for the MADRS difference. Subjects with a more pronounced decrease in depressive symptoms over time also reported a more pronounced reduction in SD (Time × MADRS Median Split *F*_4.665;157_ = 3.544, *P* = 0.004). Furthermore, all available information on depression was used to explore the impact on SD of the change in depressive symptoms as indexed by the following three dimensions: (i) observed mood and anxiety, (ii) cognitive symptoms, and (iii) neurovegetative symptoms. The decrease in depressive symptoms between baseline and week 12 was most pronounced for observed mood and anxiety symptoms (mean difference = 1.69, SD = 0.87), followed by cognitive symptoms (mean difference = 1.42, SD = 0.87). The smallest decrease was observed for neurovegetative symptoms (mean difference = 1.13, SD = 0.91). All differences were significant (*P <* 0.001). In a supplementary analysis, the ANOVA was repeated using the differences in the scores of these three depressive symptom dimensions between baseline and week 12 rather than the difference in the MADRS score. The impact on SD of the change in depressive symptoms was most pronounced for the cognitive (Time × Cognitive Symptoms *F*_4.647;155_ = 4.305, *P* = 0.001, η^2^ = 0.027) and neurovegetative symptoms (Time × Neurovegetative Symptoms F_4568_._155_ = 4.150, *P* = 0.001, η^2^ = 0.026). The smallest impact on SD was observed for change in mood and anxiety symptoms (Time × Observed Mood and Anxiety Symptoms *F_4630;155_* = 3.541, *P* = 0.005, η^2^ = 0.022).

Tests were then performed to assess the impact of age on SD. A trend towards a main effect for Age was observed (*F*_1;153_ = 3.039, *P* = 0.083), with older age being positively correlated with SD score.

**Figure 3:**
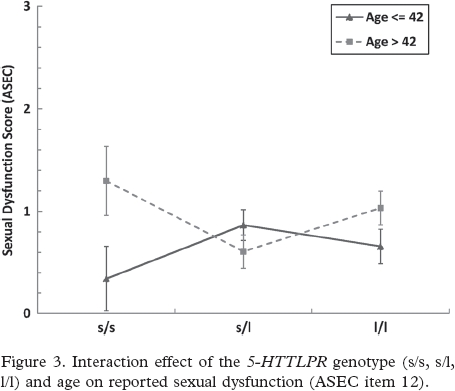
Interaction effect of the *5-HTTLPR* genotype (s/s, s/l, 1/1) and age on reported sexual dysfunction (ASEC item 12).

The final analysis tested for an association between *5-HTTLPR* genotype and SD. No main effect was detected for Genotype (*F*_2;142_ = 0.057, *P* = 0.945). Similarly, no main effect was detected for any of the following interactions: Time × Genotype (*F*_9.726;142_ = 0.812, *P* = 0.614); Medication × Genotype (*F*_2;142_ = 0.293, *P* = 0.747); or three-way Time × Medication × Genotype (*F*_9.726;142_ = 0.872, *P* = 0.557). To test for a possible interaction between age and genotype on reported SD, the continuous variable age was converted into a categorical variable by performing a median split (Median Split <42 vs. >42). The subsequent ANOVA revealed a significant interaction for Age Median Split × Genotype (*F*_2;131_ = 3.559, *P* = 0.031), with younger s/s subjects reporting less SD than older s/s subjects (see Figure 3). Again, this effect was not significantly modified by medication (Medication × Age Median Split × Genotype F_2;131_ = 0.004, *P* = 0.996).

### UKU Side Effects Rating Scale

Table II shows the frequency of SD for each UKU item. Desire dysfunction decreased significantly over time (main effect Time *F*_1.888;211_ = 24.219, *P* < 0.001). Women reported more severe desire dysfunction than men (main effect Sex *F*_1;211_ = 10.100, *P* = 0.002). This gender effect did not change over time (Time × Sex *F*_1.888;211_ = 0.048, *P* = 0.947). The decrease over time showed a trend towards being more pronounced in the escitalopram group (interaction Time × Medication *F*_1.896;209_ = 2.746, *P* = 0.068). The change in depressive symptoms had a significant impact on desire dysfunction (interaction Time × MADRS *F*_1.909;205_ = 4.691, *P* = 0.011). Subjects with a more pronounced decrease in depressive symptoms also reported a more pronounced reduction in desire dysfunction. Older subjects reported more severe desire dysfunction (main effect Age *F*_1;204_ = 5.248, *P* = 0.023).

**Table II:** Percentage of reported SD according to the *UKU* items.

			Baseline	SW 8	SW 12
Desire	Males	None	40.4	58.3	67
		Mild	22.3	22.2	16
		Moderate	26.5	12	13
		Severe	10.8	7.4	4
	Females	None	29.6	48	49.4
		Mild	16.4	18.1	20.8
		Moderate	21.8	17.2	14.3
		Severe	32.1	16.7	15.5
Erection	Males	None	86.4	88.5	86.7
		Mild	7.5	6.3	7.8
		Moderate	4.1	3.1	5.6
		Severe	2	2.1	0
Ejaculation	Males	None	92.5	85.7	87.9
		Mild	5.5	2.2	5.5
		Moderate	2.1	7.7	6.6
		Severe	0	4.4	0
Lubrication	Females	None	90.6	92.6	93.5
		Mild	3	4	2.9
		Moderate	2.6	1.1	1.4
		Severe	3.9	2.3	2.2
Orgasm	Males	None	88.5	86	90.5
		Mild	5.8	9.3	8.3
		Moderate	4.3	1.2	0
		Severe	1.4	3.5	1.2
	Females	None	77.4	84.1	84.1
		Mild	6.3	6.5	6.5
		Moderate	5	2.4	2.9
		Severe	11.3	7.1	6.5

SW, study week.

Erectile dysfunction did not change over time, and no association with study medication was found. Erectile dysfunction was more common in subjects who showed a less pronounced decrease in depressive symptoms (main effect MADRS *F*_1.63_ = 4.396, *P* = 0.040), and in subjects who were older (main effect Age *F*_1.62_ = 4.274, *P* = 0.043). Both effects were independent of time (interaction Time × MADRS *F*_1.629;63_ = 0.468, *P* = 0.588; interaction Time × Age *F*_1.625;62_ = 0.194, *P* = 0.778). Ejaculation dysfunction showed a trend towards a decrease over time (main effect Time *F*_1.832;63_ = 3.120, *P* = 0.052), and no association was found with study medication, change in depressive symptoms, or age. For vaginal lubrication dysfunction, all possible effects were non-significant.

Orgasmic dysfunction showed a trend towards a decrease overtime (main effect Time *F*_1.497;151_ = 2.856, *P* = 0.075). Women reported more severe orgasmic dysfunction than men (main effect Sex *F*_1;151_ = 3.490, *P* = 0.064). This trend did not reach significance and did not change over time (interaction Time × Sex *F*_1.497;151_ = 0.034, *P* = 0.930). Subjects with a more pronounced decrease in depressive symptoms over time also reported a more pronounced reduction in orgasmic dysfunction (interaction Time × MADRS *F*_1.522;146_ = 4.061 *P* = 0.028). No association was found with study medication or age.

### Sexual Functioning Questionnaire

Table III shows the frequency of SD for each of the SFQ subscales. Desire dysfunction significantly decreased over time (main effect Time *F*_1.000;52_ = 7.028, *P* = 0.011). Women reported more severe desire dysfunction than men (main effect Sex *F*_1;52_ = 4.239, *P* = 0.045). This difference between women and men did not change over time (interaction Time × Sex *F*_1.000;52_ = 0.257, *P* = 0.614). The decrease in desire dysfunction was marginally more pronounced in the escitalopram group (interaction Time × Medication *F*_1.000;50_ = 2.561, *P* = 0.116). Subjects with a more pronounced decrease in depressive symptoms also reported less severe desire dysfunction (main effect MADRS *F*_1;48_ = 11.259, *P* = 0.002). Older subjects reported more severe desire dysfunction than younger subjects (main effect Age (F_1;47_ = 6.421, *P* = 0.015).

**Table III:** Mean and standard deviation of reported SD according to the *SFQ* subscales.

			Baseline	SW 8	SW 12
Desire	Males	Mean	1.82	1.41	1.20
		Standard deviation	1.59	1.67	1.56
	Females	Mean	3.18	2.06	2.63
		Standard deviation	1.89	1.97	2.07
Arousal	Males	Mean	2.02	1.6	1.35
		Standard deviation	1.44	1.52	1.33
	Females	Mean	2.94	2.19	2.28
		Standard deviation	1.46	1.57	1.57
Erection	Males	Mean	1.80	1.49	1.26
		Standard deviation	1.77	1.95	1.69
Ejaculation	Males	Mean	0.6	0.25	0.45
		Standard deviation	0.9	0.55	0.77
Lubrication	Females	Mean	2.85	2.48	2.41
		Standard deviation	1.35	1.46	1.47
Orgasm	Males	Mean	1.23	1.16	0.68
		Standard deviation	1.06	1.25	1.09
	Females	Mean	2.57	2.0	2.36
		Standard deviation	1.67	1.48	1.55

SW, study week.

Arousal dysfunction significantly decreased over time (main effect Time *F*_1.000;51_ = 12.641,*P* = 0.001). Subjects with a more pronounced decrease in depressive symptoms over time also reported a more pronounced reduction in arousal dysfunction (interaction Time × MADRS *F*_1.000;47_ = 7.450, *P* = 0.009). No significant effects were found for study medication, sex, or age.

Erectile dysfunction decreased over time (main effect Time *F*_1.000;21_ = 5.469, *P* = 0.029) and older subjects reported more severe erectile dysfunction (*F*_1;18_ = 6.157,*P* = 0.023). No influence was observed for study medication or change in depressive symptoms. No significant effects were observed for ejaculation dysfunction or vaginal lubrication dysfunction.

Orgasmic dysfunction decreased over time in men but not in women (interaction Time × Sex *F*_1.000;42_ = 6.402, *P* = 0.015), and men reported less severe orgasmic dysfunction than women (main effect Sex F_1;42_ = 14.680, *P* < 0.001). All other SFQ main and interaction effects failed to reach statistical significance.

## Discussion

The primary aim of the present study was to investigate the association between the *5-HTTLPR* and SD during treatment with antidepressants with contrasting modes of action within the context of the large pharmacogenetic GENDEP study. We hypothesised that there would be a significant association with SD during treatment with the SSRI escitalopram but not during treatment with the TCA nortriptyline.

Our most prominent finding was that the majority of subjects reported no, or only mild, SD despite explicit and frequent assessment. The highest rates of SD were reported at baseline, when subjects were still medication-free, particularly in females. Over the course of treatment, reported SD showed a slight decrease. After 12 weeks of treatment, 16% of males and 24% of females reported moderate or severe SD. The decrease over time was only statistically significant in females, and was based on a pronounced decrease in reported SD within the first study week, i.e. between baseline and week 1. Of those subjects with no SD at baseline, 15.1% in the escitalopram and 16.4% in the nortriptyline group reported SD at week 8; and 14.9% in the escitalopram group and 19.7% in the nortriptyline group reported SD at week 12.

A supplementary analysis of the two more in-depth measures (UKU and SFQ) indicated that lack of desire was the most prominent SD in both men and women. This decreased over time, and escitalopram may have had a more positive effect than nortriptyline. Desire and orgasmic dysfunction were more frequent and severe in women than in men. Sexual difficulties in the other phases of the sexual cycle showed no change, or only a slight decrease, over time. This finding is plausible, since previous reports have suggested that SSRIs and TCAs cause reduced sexual desire and orgasmic dysfunction (Clayton et al. 2006a; Corona et al. 2009; Rosen et al. 1999; Serretti and Chiesa 2009; Werneke et al. 2006); however, it could also be due to the depressive disorder. Lack of sexual desire and orgasmic dysfunction are also more prevalent in women than in men in the general population (Laumann et al. 1999).

The SD rates in the present prospective and randomised sample are substantially lower than those found in two previous studies by Clayton et al. (2006b, 2007) which reported rates of 34 to 48.7% following eight weeks treatment with escitalopram. In comparison to the present study, the above reports had narrower inclusion criteria. Clayton et al. (2006b) only included subjects with (self-reported) normal sexual functioning at baseline, who had agreed to report any changes in sexual functioning, and who reported that they were sexually active at least once every 2 weeks. The authors excluded subjects with pre-existing SD, anorexia nervosa, bulimia, seizure disorders, brain injury, panic disorder, obsessive-compulsive disorder, post-traumatic stress disorder, acute stress disorder or a history of attempted suicide within the previous 6 months. The impact of these differences in study protocol on reported SD rates is unknown. It is plausible, however, that the patients’ knowledge of the explicit aim to assess SD may have enhanced their awareness of such symptoms.

The results of a multinational study that compared escitalopram with paroxetine are consistent with those of the present study (Baldwin et al. 2006). In both treatment groups, reported SD declined over the course of treatment, and differences between treatment groups were not significant, although reported SD rates were higher than in the present study. Again, the inclusion criteria were more narrowly defined. The authors argued that the decline may have been due to the dropping-out of patients with more severe SD (Baldwin et al. 2006). However, drop-outs in the present study did not differ substantially from subjects who completed the protocol in terms of reported SD. Moreover, a lower prevalence of ejaculatory problems has been reported for escitalopram compared with other SSRIs following 8 weeks of treatment (Hirschfeld and Vornik 2004). A small study (*N* = 47) reported a reduction in SD in the majority of subjects who switched from other SSRIs or serotonin-norepinephrine reuptake inhibitors to escitalopram (Ashton et al. 2005). Escitalopram is associated with lower rates of treatment-withdrawal, and fewer discontinuation symptoms, than paroxetine (Baldwin et al. 2006, 2007).

It is important to acknowledge that SD is widespread in the general population. Estimates of 11% for frequent or severe SD and 69% for infrequent or mild difficulties have been reported. Men predominantly report ejaculatory problems, with overall rates of between 10 and 20%, whereas lack of sexual desire is most prevalent in women, with rates of between 20 and 25%. Women also report higher rates of orgasm dysfunction than men (Laumann et al. 1999; Montgomery et al. 2002; Derogatis and Burnett 2008; Laumann et al. 2009; Christensen et al. 2010). In the present study, the SD rates reported after 12 weeks of antidepressant treatment (16% in males and 24% in females) only slightly exceed the SD rates in the general population.

Given the rather low rates of reported SD, it is not surprising that we failed to detect a major influence of study medication or the *5-HTTLPR* on reported SD or its time course in the analysis of the main outcome measure. No significant differences in reported SD were observed between patients receiving escitalopram and those receiving nortriptyline.

A more pronounced decrease in reported SD was found in subjects whose depression improved substantially. This is consistent with previous findings for escitalopram, duloxetine, nortriptyline, and sertraline, and suggests that SD is influenced by the remission of depression and may not be a side-effect of antidepressant treatment (Lanza di Scalea et al. 2009). The effect was similar for the three depressive symptom dimensions “observed mood and anxiety", “cognitive symptoms", and “neurovegetative symptoms". This may be due to the high correlation between the changes in depressive symptoms over time across the three dimensions. Observed mood and anxiety symptoms showed the most pronounced improvement and neurovegetative symptoms the least. The effects of study medication on the improvements in each of these three symptom dimensions have been described and discussed elsewhere (Uher et al. 2009c).

We also observed an influence of age on reported SD. Younger subjects reported less SD than older subjects particularly for desire and erection difficulties. This finding is plausible, since age is an established risk factor for SD (Phanjoo 2000; Derogatis and Burnett 2008; Corona et al. 2010).

Finally, we found no influence of *5-HTTLPR* genotype on reported SD on average, over the time course of treatment, or in interaction with the study medication. However, our data suggest a possible influence of age on the association between the *5-HTTLPR* genotype and reported SD. The influence of age on sexual function may be more pronounced in s/s subjects than in patients with the genotype s/1 or 1/1. One previous report found that the s/s genotype was associated with less SD than the 1/1 genotype during SSRI treatment, although this study did not report any influence of age (Bishop et al. 2009). However, the age distribution of the Bishop et al. sample (2009) differed significantly (*t* = 11.70, *P <* 0.01) from that of the present study. In the present sample, subjects with genotype data were between 19 and 72 years of age, and the mean age was 42.11 years. The study by Bishop et al. (2009) included relatively young patients of between 18 and 40 years of age (mean of 29.2 years) in order to minimise the confounding effect of age on the presence of SD. The lower proportion of older subjects may explain why Bishop et al. (2009) found no influence of age on the association between the *5-HTTLPR* genotype and reported SD.

The present study met several of the methodological quality criteria for the evaluation of antidepressant related SD (Montgomery et al. 2002; Schweitzer et al. 2009). These included: (i) a randomised and prospective design; (ii) comparisons between different treatments; (iii) a baseline assessment; (iv) the use of defined diagnostic criteria for depression; (v) the application of reliable and validated rating scales for the assessment of SD in men and women; (vi) direct assessment of depression and sexual function before and during treatment; and (vii) consideration of the possible effects of degree of remission and gender on the outcome variables. However, the study had several limitations. Firstly, we did not control for comorbid illness or concomitant medications that might affect normal sexual function. A previous study, which involved neither random assignment nor baseline control, controlled for these factors and compared several different antidepressants. This found the highest prevalence rates for SD during treatment with the SSRI citalopram (Clayton et al. 2002). We did, however, exclude individuals who had a contraindication to both study medications, who were currently being treated for a somatic disease using potentially depressogenic medications, or who were using other psychotropic medications, with the exception of the occasional use of hypnotics. A second very important limitation is that patients were not asked whether they had a sexual partner or questioned about their actual sexual activity during the course of the study (sexual intercourse, sexual fantasies, or other sexual activities). As already mentioned, Clayton et al. (2006b) only included subjects who had normal orgasmic function and who were sexually active. Future comparative well-designed randomised controlled trials of SD during antide-pressants treatment for depression should include sexually active subjects, or at least involve enquiry about sexual activity, to determine the clinical significance of differences in SD (Schweitzer et al. 2009). Thirdly, the study was not double-blind, with in most cases in fact both the treating psychiatrist and the patient knowing which medication was being prescribed. However, knowledge of the possibility of higher SD rates during SSRI treatment is likely to have resulted in increased, rather than decreased, monitoring and awareness of these symptoms by patients. A further limitation is that our assessment of SD did not include any evaluation according to the detailed DSM-IV criteria for SD (Haberfellner 2007). Finally, our genetic analysis was limited to the *5-HTTLPR,* and it therefore remains unclear whether other polymorphisms in the serotonin transporter gene influence the development of SD.

In summary, the present study of a large multi-centre European sample found no interaction effect for *5-HTTLPR* genotype and two antidepressants with contrasting primary modes of action on reported SD. In addition, no differential influence of study medication per se was found, with the exception of a possible beneficial effect on desire dysfunction for escitalopram compared to nortriptyline. The rate of reported SD was rather low, with only minor changes or improvements being reported during the course of treatment, despite the application of three different measurement tools. We identified an influence of change in depressive symptoms and age on reported SD. The unexpectedly low rate of SD reported during treatment with both medications is a noteworthy finding of the present study, which may have important implications for clinical practice.

## Statement of Interest

Strohmaier, Wüst, Uher, Mors, Hauser, Souery, Zobel, Dernovsek, Streit, Schmäl, Kozel, Placentino, and Rietschel have no conflicts of interests. Henigsberg has participated in clinical trials sponsored by pharmaceutical companies including GlaxoSmithKline and Lundbeck; Henigsberg has received honoraria for participating in expert panels from pharmaceutical companies including Lundbeck. Souery is a member of national advisory boards for Astra-Zeneca, Bristol-Myers Squibb, Eli Lilly, and Lundbeck. Aitchison, Farmer, and McGuffin have received consultancy fees and honoraria for participating in expert panels for pharmaceutical companies including Lundbeck, GlaxoSmithKline, and Bristol-Myers Squibb and Otsuka Pharmaceuticals Limited. EudraCT clinical trials registrynumber,2004-001723-38(http://eudract.emea.europa.eu) and International Standard Randomized Controlled Trials registry number, ISRCTN 03693000 (http://www.controlled-trials.com).
